# Seed Halopriming Improves Salinity Tolerance of Some Rice Cultivars During Seedling Stage

**DOI:** 10.1186/s40529-022-00354-9

**Published:** 2022-07-25

**Authors:** Anik Hidayah, Rizka Rohmatin Nisak, Febri Adi Susanto, Tri Rini Nuringtyas, Nobutoshi Yamaguchi, Yekti Asih Purwestri

**Affiliations:** 1 Biotechnology Study Program, The Graduate School, Universitas Gadjah Mada, Jl. Teknika Utara, Sleman, Yogyakarta, 55281 Indonesia; 2 Biochemistry Laboratory, Department of Tropical Biology, Faculty of Biology, Universitas Gadjah Mada, Jl. Teknika Selatan, Sekip Utara, Yogyakarta, 55281 Indonesia; 3 Research Center for Biotechnology, Universitas Gadjah Mada, Jl. Teknika Utara, Sleman, Yogyakarta, 55281 Indonesia; 4 Agricultural Environmental Division, Indonesian Agency For Agricultural Research and Development, Jl. Raya Jakenan-Jaken Km. 5, Central Java 59182 Pati, Indonesia; 5 Plant Stem Cell Regulation and Floral Patterning Laboratory, Graduate School of Biological Sciences, Nara Institute of Science and Technology, Ikoma, Japan

**Keywords:** Salinity tolerance, Seed halopriming, Standard Evaluation System for Rice (SES), Transporter genes, Seedling stage

## Abstract

**Background:**

Saline land in coastal areas has great potential for crop cultivation. Improving salt tolerance in rice is a key to expanding the available area for its growth and thus improving global food security. Seed priming with salt (halopriming) can enhance plant growth and decrease saline intolerance under salt stress conditions during the subsequent seedling stage. However, there is little known about rice defense mechanisms against salinity at seedling stages after seed halopriming treatment. This study focused on the effect of seed halopriming treatment on salinity tolerance in a susceptible cultivar, IR 64, a resistant cultivar, Pokkali, and two pigmented rice cultivars, Merah Kalimantan Selatan (Merah Kalsel) and Cempo Ireng Pendek (CI Pendek). We grew these cultivars in hydroponic culture, with and without halopriming at the seed stage, under either non-salt or salt stress conditions during the seedling stage.

**Results:**

The SES scoring assessment showed that the level of salinity tolerance in susceptible cultivar, IR 64, and moderate cultivar, Merah Kalsel, improved after seed halopriming treatment. Furthermore, seed halopriming improved the growth performance of IR 64 and Merah Kalsel rice seedlings. Quantitative PCR revealed that seed halopriming induced expression of the *OsNHX1* and *OsHKT1* genes in susceptible rice cultivar, IR 64 and Merah Kalsel thereby increasing the level of resistance to salinity. The expression levels of *OsSOS1* and *OsHKT1* genes in resistant cultivar, Pokkali, also increased but there was no affect on the level of salinity tolerance. On the contrary, seed halopriming decreased the expression level of *OsSOS1* genes in pigmented rice cultivar, CI Pendek, but did not affect the level of salinity tolerance. The transporter gene expression induction significantly improved salinity tolerance in salinity-susceptible rice, IR 64, and moderately tolerant rice cultivar, Merah Kalsel. Induction of expression of the *OsNHX1 and OsHKT1* genes in susceptible rice, IR 64, after halopriming seed treatment balances the osmotic pressure and prevents the accumulation of toxic concentrations of Na^+^, resulting in tolerance to salinity stress.

**Conclusion:**

These results suggest that seed halopriming can improve salinity tolerance of salinity-susceptible and moderately tolerant rice cultivars.

## Background

Salinity is a major problem in the production of cereal crops throughout the world (Ibrahim 2016; Reddy et al. 2017; Walia et al. 2007). Continual salt intrusion as a result of global warming (Wang et al. 2018) and irrigation practices (Reddy et al. 2017) increases soil salinity. Rice (*Oryza sativa* L.) is sensitive to salinity (Yoshida et al. 1976), especially during the seedling stage (Sakina et al. 2016; Zhao et al. 2014). Although rice is sensitive to salt, it is the only cereal crop that is recommended for cultivation in saline land. This is due to the ability of some rice plants to grow well in stagnant water and to leach salts from the surface of the soil to the soil beneath. By diluting the salts, the plants increase the availability of nutrients such as iron, manganese, nitrogen, phosphorus, and silicon, as well as conserve nitrogen and reduce water stress (Lafitte et al. 2004). Saline land in coastal areas has great potential for crop cultivation and supporting food security, so engineering high-yield, salt-tolerant rice genotypes is an important goal. The susceptibility or tolerance of rice plants to salinity stress is determined by the coordinated action of multiple stress-responsive genes, which also associate with other components of stress signal transduction pathways (Reddy et al. 2017).

Seed priming is one option for alleviating the effects of salt stress and preserving plant metabolism under saline conditions. Seed priming increases the natural tolerance potential of the seed to abiotic stress, representing a value-added solution that can be implemented at an early stage of rice production to induce mechanisms that help the plants tolerate salinity stress during later growth (Yang et al. 2018). Many researchers have used conventional plant breeding to develop salt-tolerant cultivars, but this requires high technology, complicated procedures, and long timeframes to obtain one tolerant cultivar (Breseghello 2013). Seed priming is a simple and promising technique for improving plants’ stress tolerance that does not require producing a genetically modified organism (Moreno et al. 2018). Different seed priming treatments in wheat (*Triticum aestivum*) seeds increase salt tolerance, and osmopriming techniques with CaCl_2_ are the most effective treatments for obtaining higher grain yields (Jafar et al. 2012). The use of *n*-Fe_2_O_3_ as a pre-sowing seed treatment can increase the germination and growth of sorghum (*Sorghum bicolor*) seeds and protect the plants from salt stress (Maswada et al. 2018). Evaluating the effectiveness of priming treatments in *Chenopodium quinoa* and *Amaranthus caudatus* seeds to improve germination under salt stress showed that seed hydropriming and osmopriming caused significant improvements in germination velocity and uniformity, reflected in high final germination percentages, high germination indexes, and reduced mean germination times under salinity. *C. quinoa* had a higher tolerance to salinity than *A. caudatus* during seed germination (Moreno et al. 2018).

The transmembrane movement of Na^+^ and K^+^ in plants is mediated by several types of transporters and/or channels, and many transporters have been implicated in Na^+^ exclusion from leaves (Wangsawang et al. 2018). These include members of the high-affinity K^+^ transporter (*HKT*) family, such as OsHKT2;1 (OsHKT1) and OsHKT2;4, which are expressed in the outer part of the root and in the root hairs and may provide entry points for Na^+^ into plant roots from the soil (Wangsawang et al. 2018). By contrast, *O. sativa SOS1* (*OsSOS1*) is implicated in the conservation of the salt-sensitive pathway in rice (Martı´nez-Atienza et al. 2007). In addition to Na^+^ exclusion, plants may avoid toxic Na^+^ accumulation in the cytosol by sequestering excess Na^+^ in vacuoles, which is mediated by the Na^+^/H^+^ antiporter (NHX1) localized in the vacuolar membranes (Wangsawang et al. 2018).

Hydroponic culture is a reliable method of assessing the response of genotypes to salt stress (Sakina et al. 2016). Experiments evaluating responses to salinity stress in different plant species using hydroponic culture have been conducted in many plants, including rice (Manimaran et al. 2017; Sakina et al. 2016; Walia et al. 2007; Wang et al. 2016) and barley (*Hordeum vulgare*) (Widodo et al. 2009). All of these studies used Yoshida's solution as a nutrient. Yoshida’s nutrient solution, which is routinely used for growing rice plants in hydroponic culture, consists of macronutrients and micronutrients needed by plants to grow well (Yoshida et al. 1976).

Pigmented rice is widely consumed because of its high nutritional value and antioxidant contents, which benefit human health. In addition, several Indonesian black rice cultivars are reportedly resistant to bacterial blight disease (Sutrisno et al. 2018). The possible involvement of antioxidant genes in drought and salinity stress tolerance in leaves of Indonesian black rice (*Oryza sativa* Cv. Cempo Ireng Pendek (CI Pendek)) seedlings has been studied (Purwestri and Refli 2016). Dismutation of superdioxide radicals and biosynthesis of reduced ascorbic acid in the gluthationine–ascorbate cycle within cells are lower in seedlings under drought stress, so the oxidative damage to seedlings under drought is higher than that under salinity, indicating that CI Pendek is more resistant to salinity stress than drought stress. Our preliminary study showed that Indonesian black rice (CI Pendek) and red rice (Cv. Merah Kalimantan Selatan (Merah Kalsel)) will grow on media with concentrations of up to 200 mM NaCl after seed priming treatment.

Several priming techniques are available; depending on the priming agents, they are classified as hydropriming, osmopriming, halopriming, hormone priming, hardening, solid matrix priming, humidification and stratification, or thermal shock. The first four approaches are the most commonly used; these techniques are simple and easy, for example, soaking seeds in water or a solution containing inorganic salt, sugar, or hormones followed by air drying before sowing. This improves the growth, emergence, and yield of the crop (Nawaz et al. 2013; Paparella et al. 2015). In this study, we used a halopriming technique combining NaCl, CaCl_2_, KCl, KNO_3_, and H_2_O_2_ to induce salinity tolerance in (i) Indonesian pigmented rice (CI Pendek and Merah Kalsel), (ii) salinity-tolerant rice (*O. sativa* Cv. Pokkali (Pokkali)), and (iii) salinity-susceptible rice (*O. sativa* Cv. IR 64 (IR 64)). The objectives of this study were to (a) determine the effect of seed halopriming on the salinity resistance of rice seedlings with different tolerance levels; (b) identify morpho-physiological changes of rice plants in the early growth stage after halopriming treatment of seeds; and (c) study the molecular mechanism of salinity resistance based on transporter gene expression in rice seedlings after seed halopriming treatment.

## Materials and methods

### Rice materials

Two Indonesian pigmented rice cultivars, Cv. Merah Kalsel and Cv. CI Pendek, were obtained from the germplasm collection at Gadjah Mada University in Indonesia. The seeds for the other two cultivars, Pokkali and IR 64, were obtained from the Indonesian Center for Rice Research (ICRR).

### Methods

#### Seed priming treatment

Four selected rice cultivar seeds were surface sterilized by soaking in 10% sodium hypochlorite solution for 15 min, followed by washing three times with distilled water, each for 15 min. The sterilized seeds were then soaked in a solution consisting of 100 mM NaCl, 2.2% CaCl_2_, 2.2% KCl, 2.2% KNO_3_, and 50 mM H_2_O_2_ for 48-h priming. NaCl, CaCl_2_, KCl, KNO_3_, and H_2_O_2_ of 99.9% purity and ultra-pure water were used to adjust concentrations. The resulting seeds were dried back to their original moisture content before use. Unprimed dry seeds were used as a control.

#### Plant growth condition and salinity treatment

Unprimed and primed seeds were imbibed in distilled water at 27/28 °C for 12 h in dark conditions. For germination and seedling establishment, the seeds were placed on moist filter paper over a Petri dish for 7 days until the second or third leaf of the seedlings emerged in controlled conditions at 30/27 °C day/night with a photoperiod regime of 12/12 h day/night. The resulting sprouts were watered with distilled water every day. Three to five 7-day-old rice seedlings were transplanted to a black seed tray (size: 28 cm × 10 cm with a 21 hole), so each tray could accommodate 63–105 seedlings. The trays were placed in plastic containers (35 × 30 × 15 cm) filled with Yoshida's solution (9.14% NH_4_NO_3_, 4.03% NaH_2_PO_4_·2H_2_O, 7.14% K_2_SO_4_, 8.86% CaCl_2_, 32.40% MgSO_4_·7H_2_O, 0.15% MnCl·4 H_2_O, 0.0074% (NH_4_)_6_·MO_7_O_24_·4H_2_O, 0.0934% H_3_BO_3_, 0.0035% ZnSO_4_·7H_2_O, 0.0031% CuSO_4_·5H_2_O, 0.77% FeCl_3_·6H_2_O, 1.19% C_6_H_8_O_7_·H_2_O, and 5% H_2_SO_4_). The pH of the nutrient solutions was maintained between 5.0 and 5.5 with 2 N HCl or 2 N NaOH throughout the growth period as described by Yoshida et al. (1976).

The seedlings were grown in an environmentally controlled greenhouse in the Research Center for Biotechnology, Universitas Gadjah Mada, in Indonesia at 25–32 °C with a 12-h light/12-h dark photoperiod. The nutrient solution was renewed every 7 days, and plants were doused with distilled water daily as compensation for loss of water due to evapotranspiration. Salinity treatments were performed on 21-day-old seedlings by adding NaCl to the nutrient solution until a final concentration of 200 mM NaCl or an electrical conductivity (ECw) of 21.1 dS m^–1^ was reached. The non-saline control (fed with Yoshida solution only) had an ECw of 1.1 dS m^–1^ (Bado et al. 2016). The experiments had a split-split plot design with three replicates. The treatment groups were (i) without priming (unprimed) and non-stressed; (ii) unprimed and stressed with 200 mM NaCl solution; (iii) primed and non-stressed, and (iv) primed and stressed with 200 mM NaCl solution for each rice cultivar.

#### Evaluation of salt stress symptoms

Salt stress was evaluated based on visual symptoms according to the Standard Evaluation System (SES) score for rice used by the International Rice Research Institute/IRRI (Bado et al. 2016; Gregorio et al. 1997; Wang et al. 2016; Wangsawang et al. 2018) (see Table 1). This scoring discriminates the susceptible from the tolerant and the moderately tolerant genotypes. Plants were scored between 1 and 7 days after salinization. During this time, susceptible genotypes could be distinguished but tolerant genotypes could not be readily identified from the moderately tolerant genotypes. After 7 days salinization, there will be a clear distinction among the tolerant, moderately tolerant, and susceptible genotypes (Gregorio et al. 1997).

**Table 1:** Modified standard evaluation system (SES) score of visual salt injury at seedling stage

SES score	Visual observation	Tolerance
1	Normal growth, no leaf symptoms	Highly tolerant
3	Nearly normal growth, but leaf tips or a few leaves whitish and rolled	Tolerant
5	Growth severely retarded; most leaves rolled; only a few are elongating	Moderately tolerant
7	Complete cessation of growth; most leaves dry; some plants dying	Susceptible
9	Almost all plants dead or dying	Highly susceptible

#### Measurement of chlorophyll and relative water content

Total chlorophyll content (CC) was measured for 7 days after salt treatment using a chlorophyll meter (Konica Minolta SPAD 502 Plus, Japan). Relative water content (RWC) was measured according to the methods described by Wu et al. (2018). RWC was calculated as follows:

RWC (%) = (FW − DW)/(TW − DW) × 100. Plant fresh weight (FW) was measured immediately after harvest. The plants were subsequently soaked in deionized water for 8 h at 4 °C. Then, the plants were quickly weighed to determine the turgid weight (TW), and their dry weight (DW) was measured after oven drying at 105 °C for 10 min followed by 80 °C for 24 h.

#### Measurement of plant growth responses

Ten plants of each rice cultivar were harvested from the pots after completion of the experiment. The root length and plant height were measured. Fresh samples were then oven dried at 80 °C for 72 h and DW was measured separately (Manimaran et al. 2017).

#### *Determination of Na*^+^*and K*^+^*ion content*

Root and leaf tissues from each individual plant were harvested 0, 4, and 7 d after stress treatment. The root and leaf samples were finely ground into powder after drying in an oven. The Na^+^ and K^+^ ion contents were quantified according to Manimaran et al. (2017), with minor modification. Dried leaf and root samples (500 mg) were placed in digestion tubes containing 5 ml of a nitric acid and perchloric acid (5:1, v/v) mixture; the tubes were incubated overnight. The next day, the tubes were subjected to 8 h of digestion at 175 °C with gradual increases in the heat until 300 °C was reached, to allow the mixture to clear. The digested liquid was cooled overnight, followed by filtering through Whatman no. 1 filter paper. Then, the volume was brought to 50 ml with deionized water. Sodium and potassium concentrations were analyzed using an atomic absorption spectrophotometer (AAS, Varian-240 FS). Ion concentrations in each sample were estimated using Na^+^ and K^+^ standard curves.

#### Expression analysis of transporter genes

Root and leaf tissues from each individual plant were harvested at 0, 6, and 24 h after priming and immediately frozen in liquid nitrogen. Then, the tissues were ground into a powder using a mortar and pestle under liquid nitrogen. RNA was isolated using an RNeasy Plant Mini Kit (Qiagen). The RNA concentration was determined using a Nanodrop spectrophotometer. The primers for the transporter genes (*OsSOS1, OsNHX1,* and *OsHKT1*) were designed using the online Primer3 0.4.0 software (http://bioinfo.ut.ee/primer3-0.4.0/) based on the *O. sativa* Japonica Group sequence data (Table 1). The RNA (1 µg) was subjected to cDNA synthesis using a Superscript III First-Strand Synthesis System for reverse transcription (Invitrogen). Reverse transcription-quantitative polymerase chain reaction (RT-qPCR) was performed using SYBR Green Mastermix (Bio-Rad). The reaction mixture contained 5 µl of SYBR Green Mastermix, 0.75 µl of forward primer, 0.75 µl of reverse primer, 1 µl of cDNA, and 2.5 µl of nuclease-free water. RT-qPCR was performed with the following cycles: an initial incubation at 95 °C for 30 s, followed by 40 cycles of denaturation at 95 °C for 10 s and extension at 55 °C for 40 s. Relative expression levels of the gene transcripts were calculated using the 2^−∆∆CT^ method (Livak and Schmittgen 2001). The *UBIQUITIN* gene was used as an internal control to normalize gene expression (Sutrisno et al. 2018). The sequences of the primers used are listed in Table 2.

**Table 2:** Primers used for RT-qPCR

Gene	Primer sequences (5′3′)	Number of bases	% GC content	*T*_m_ (°C)	Product size (kb)
*OsSOS1*	F: acgcaaggcaatagaagagg	20	50.00	59.48	164
	R: ttggctggtccaacaattac	20	45.00	58.48	
*OsNHX1*	F: cgggatgattggtttgttct	20	45.00	59.79	128
	R: cccgccaactaaagatggta	20	50.00	59.95	
*OsHKT1*	F: gctcaaggccttcacaaaag	20	50.00	59.99	152
	R: ggcccaattagaaacctgaa	20	45.00	59.02	
*UBIQUITIN*	F: cacaagaaggtgaagctcgc	20	55.00	62.00	183
	R: ctctctggttgtagacgtagg	21	52.00	64.00	

*T*_m_: melting temperature

#### Statistical analysis

Statistical analyses were performed using SAS 9.1 for Windows (SAS Institute, Cary, NC, USA). Analysis of variance (ANOVA) was carried out independently for each measurement using the GLM (general linear model) procedure of SAS. The standard errors are shown as an estimate of variability. Differences between the means were compared by least significant difference (LSD) at *p* < 0.05 and *p* < 0.01. The data are presented as the means and standard error (SE) of three replicates.

## Results

### Salt stress symptoms

The salinity tolerance of each rice cultivar was scored after seed priming treatment based on the SES (Gregorio et al. 1997). Pokkali was used as a positive control for salinity tolerance, whereas IR 64 was used as a salinity-susceptible control (Bado et al. 2016; Gregorio et al. 1997). IR 64 was also used to examine whether the seed priming technique used was able to increase salinity resistance in a salinity-sensitive rice cultivar. Salt treatment with up to 200 mM NaCl increased the salinity tolerance of Pokkali, a salinity-tolerant rice cultivar. Based on this, the 200 mM NaCl treatment was used for further study.

The initial signs of salt stress damage were observed in the oldest leaves, which started to desiccate and roll inward (Table 3). Signs of damage were observed in the unprimed IR 64 rice cultivar on the first day after 200 mM NaCl treatment. Three days after the treatment, signs of salt stress damage also appeared in unprimed Merah Kalsel. Four days after the treatment, the oldest leaves of primed CI Pendek started to desiccate and roll inward. Five days after the treatment, unprimed Pokkali, unprimed CI Pendek, primed Merah Kalsel, and primed IR 64 showed signs of damage due to salt stress. Primed Pokkali seedlings looked nearly normal at 7 days after treatment.

**Table 3:** Priming reduced salt damage in most tested cultivars

Cultivar	Seed priming treatment	Visible damage to the oldest leaves
Pokkali	Unprimed	5 days after stress
	Primed	No damage by 7 days after stress
CI Pendek	Unprimed	5 days after stress
	Primed	4 days after stress
Merah Kalsel	Unprimed	3 days after stress
	Primed	5 days after stress
IR 64	Unprimed	1 day after stress
	Primed	5 days after stress

**Fig. 1:**
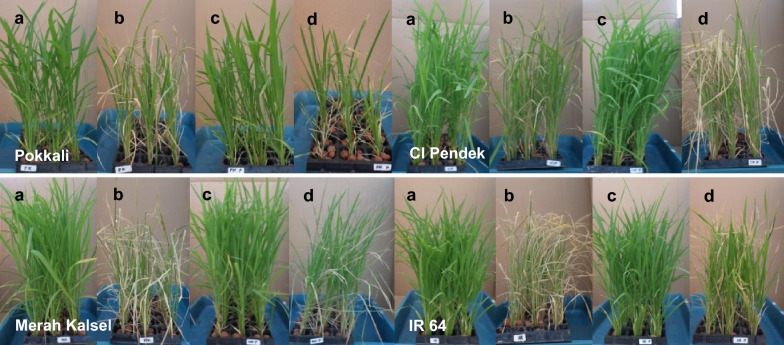
Most of the tested cultivars were less affected by salinity stress after priming. For each cultivar, an example of the following treatment groups is shown: (from left to right) **a** unprimed and non-stressed, **b** unprimed and stressed with 200 mM NaCl solution, **c** primed and non-stressed, and **d** primed and stressed with 200 mM NaCl solution

Scoring was performed 7 days after salinization (Table 4). The SES scores of three cultivars decreased after seed priming; CI Pendek was the exception. Pokkali had the lowest SES scores for both unprimed and primed seedlings (Table 4), which is consistent with its phenotype; the damage levels of these two groups of seedlings appeared to be almost the same (Fig. 1; Table 4). Unprimed CI Pendek and primed CI Pendek had similar SES scores and showed similar phenotypes under salinity stress (Fig 1; Table 4). Unprimed Merah Kalsel exhibited growth retardation, and most of its lower leaves rolled. Furthermore, some of the oldest leaves in unprimed Merah Kalsel dried up, and only the two youngest leaves remained green (Fig 1). Because of these phenotypic changes, unprimed Merah Kalsel was assessed as moderately tolerant. After salinity stress, primed Merah Kalsel had a 3.6 ± 0.97 SES score and grew better than its unprimed counterpart (Fig 1; Table 4). IR 64 is a highly salinity-susceptible cultivar (Bado et al. 2016; Gregorio et al. 1997). Consistent with these reports, the SES score of unprimed IR 64 was 8.4 ± 0.97. Surprisingly, priming greatly increased the salinity tolerance of IR 64 (Fig 1; Table 4).

**Table 4:** Seed priming greatly increased salinity tolerance of IR 64 seedlings, as measured by SES score 7 days after salinity stress

Cultivar	Seed priming treatment	SES score	Level of salinity tolerance
Pokkali	Unprimed	2.6 ± 0.84	Tolerant
	Primed	2.4 ± 0.97	Tolerant
CI Pendek	Unprimed	3.4 ± 0.84	Tolerant
	Primed	3.8 ± 1.03	Tolerant
Merah Kalsel	Unprimed	4.6 ± 0.84	Moderately tolerant
	Primed	3.6 ± 0.97	Tolerant
IR 64	Unprimed	8.4 ± 0.97	Highly sensitive
	Primed	3.6 ± 0.97	Tolerant

The Standard Evaluation System (SES) for rice was used to assess visual salt damage in seedlings at 7 days after salinization (200 mM NaCl). SESscores: 1 (highly tolerant), 3 (tolerant), 5 (moderately tolerant), 7 (sensitive) and 9 (highly sensitive). Values are the means of ten seedlings ± SE

### Chlorophyll content and relative water content in leaves

**Fig. 2:**
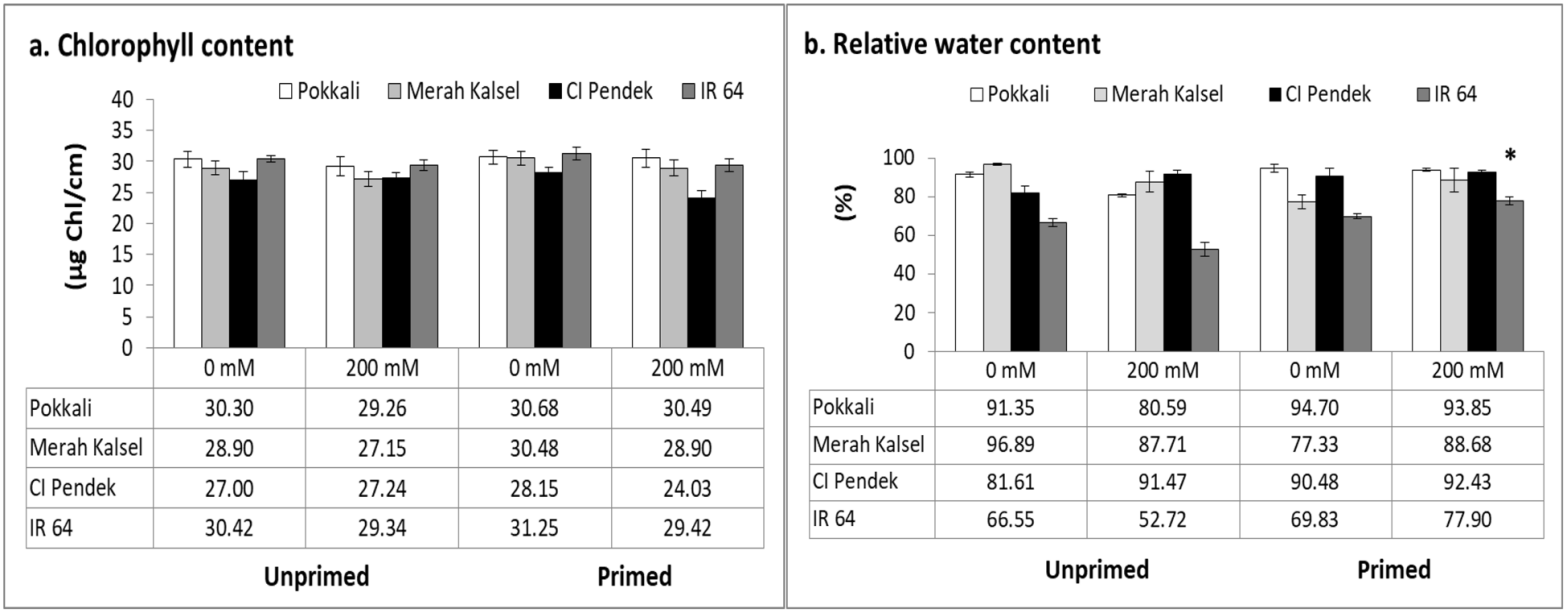
Effect of salinity stress on agronomic parameters of rice plants. **a** Chlorophyll content, **b** relative water content (RWC). Chlorophyll contents were measured for 7 days after salinity treatment; relative water content in the whole plant with and without priming was measured 7 days after salinity stress. Values are means of three replicates ± SE. *p < 0.05, **p < 0.01, LSD test

To quantify damage levels after salinity stress with and without priming, the total chlorophyll content (CC) in leaves was examined. CCs in primed Pokkali and Merah Kalsel were higher than those in unprimed Pokkali and Merah Kalsel under salinity (Fig 2a). CCs of IR 64 leaves were similar in unprimed and primed treatments (Fig 2a). By contrast, CCs of primed CI Pendek plants were lower than those of unprimed CI Pendek plants under salinity (Fig. 2a).

As with CC quantification, water content of the whole plant is often measured to determine stress levels of a plant (Wangsawang et al. 2018). There was no change in relative water content (RWC) in unprimed Merah Kalsel or salinity-tolerant Pokkali and CI Pendek, regardless of salinity stress (Fig 2b). Without priming, salinity stress decreased the RWC in IR 64 (Fig 2b). Seed priming treatment led to a significantly increased RWC in IR 64 (*p* < 0.01; LSD test; Fig 2b). Although the extent of increased RWC was different between rice cultivars, similar effects were observed in primed Pokkali, Merah Kalsel, and CI Pendek (Fig 2b). These results suggest that seed priming increases the ability of rice plants to maintain their RWC.

### Plant growth responses

**Fig. 3:**
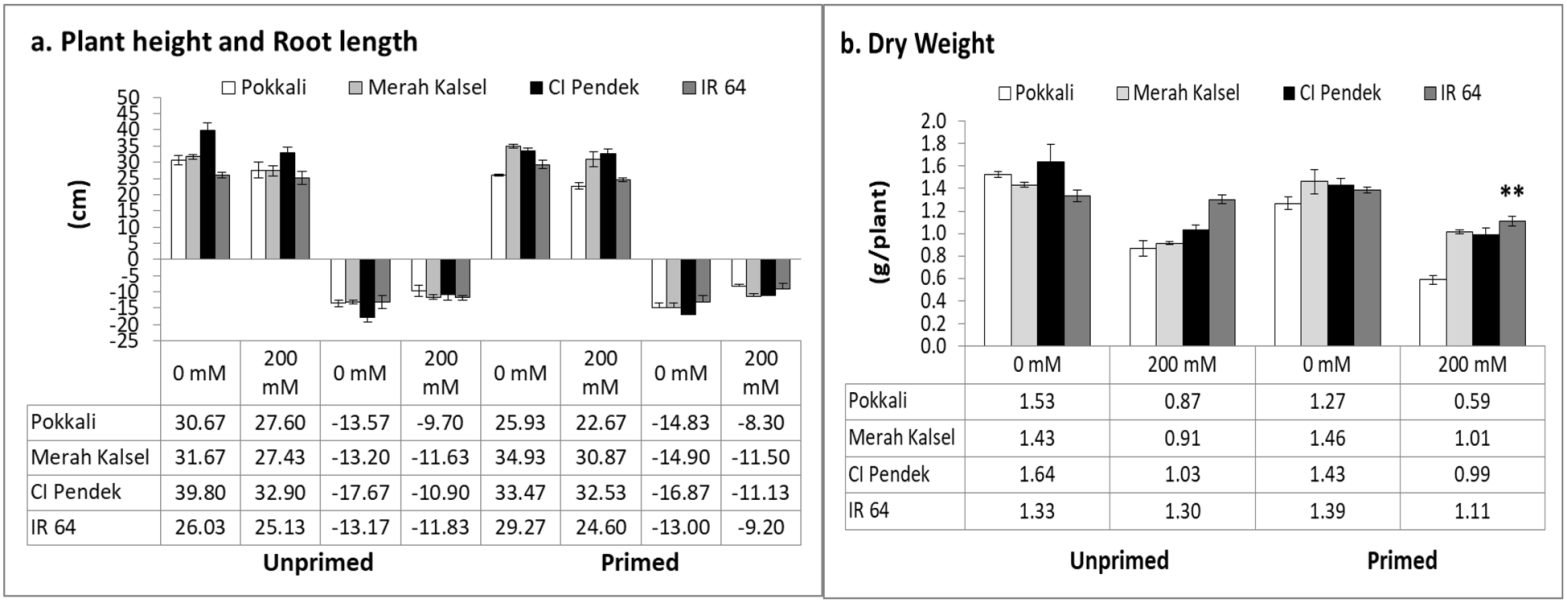
Effect of salinity stress on agronomic parameters of rice plants. **a** Plant height and root length and **b** dry weight of whole plant with and without priming were measured 7 days after salinity stress. Values are means of three replicates ± SE

To understand the effect of salinity stress on plant growth with and without priming, the plant height, root length, and dry weight biomass were examined (Fig 3). Overall, salinity stress led to decreased shoot length, root length, and dry biomass in seedlings of all four rice cultivars, regardless of priming. In unprimed controls, salinity stress led to decreased plant height, root length, and dry weight in all four rice cultivars. Priming with NaCl partially rescued plant height and root length after salinity stress in Merah Kalsel and IR 64 compared with equivalent unprimed controls (Fig 3a). Salinity stress decreased plant height and root length in primed Pokkali and CI Pendek compared with unprimed controls (Fig 3a). Compared with the control plants, decreases in dry weight in unprimed IR 64 were the most severe of all the rice cultivars. When salinity sensitive-IR 64 seeds were primed, the reduction in dry weight was rescued. Furthermore, only slight decreases in dry weight in the other three primed rice cultivars were observed (Fig 3b). Although size changes in response to salinity in primed and unprimed plants differed between rice cultivars, seed priming sufficiently increased the level of salinity tolerance to affect dry weight.

### Na^+^ and K^+^ ion content

**Fig. 4:**
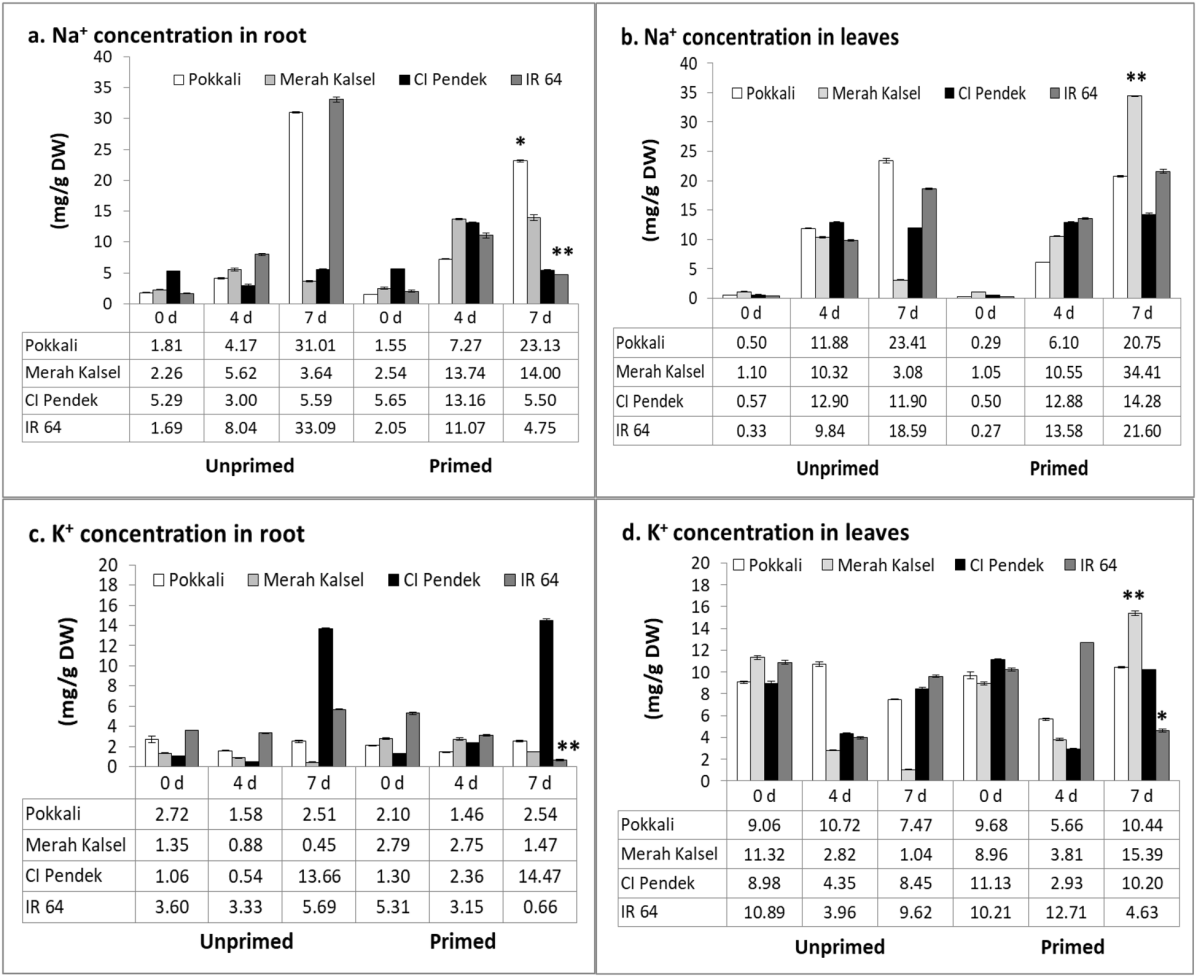
Concentration of Na^+^ and K^+^ ions of rice plants after 7 days exposed to salinity stress. **a**, **c** Na^+^ and K^+^ ion concentration in roots. **b**, **d** Na^+^ and K^+^ ion concentration in leaves were measured at 0, 4, and 7 days of salinity treatment (200 mM NaCl). Values are means of three replicates ± SE. *p < 0.05, **p < 0.01, LSD test. DW: dry weight

To test whether ion transport differed between primed and unprimed seedlings after salinity stress, Na^+^ and K^+^ accumulations were examined. Seed priming led to increased Na^+^ concentrations in Merah Kalsel roots but decreased Na^+^ concentrations in the roots of the other rice cultivars at 7 days after salinity treatment (Fig 4a). Furthermore, Na^+^ concentrations in primed IR 64 (*p* < 0.01, LSD test) and primed Pokkali (*p* < 0.05, LSD test) roots significantly decreased under salinity. Seed priming significantly increased Na^+^ accumulations in Merah Kalsel leaves under salinity treatment (*p* < 0.01, LSD test) (Fig 4b).

Seed priming also affected K^+^ concentrations in roots and leaves. Under salinity stress, K^+^ concentrations in Pokkali roots decreased at 4 days after salt treatment (Fig 4c). But K^+^ concentrations in Pokkali roots returned to or surpassed their original levels in both unprimed and primed seedlings at 7 days after salinity stress (Fig 4c). K^+^ concentrations in unprimed and primed Merah Kalsel roots decreased at 7 days after salinity treatment (Fig 4c). Both unprimed and primed CI Pendek roots showed the highest K^+^ concentrations at 7 days after salt treatment (Fig 4c). Seed priming treatment in IR 64 resulted in decreased K^+^ concentrations in roots (Fig 4c). K^+^ concentration decreased at 7 days after salinity stress in unprimed Pokkali, unprimed Merah Kalsel, and primed IR 64 leaves. K^+^ concentration decreased at 4 days after salinity stress in primed Pokkali, primed Merah Kalsel, unprimed and primed CI Pendek, and unprimed IR 64 leaves but increased at 7 days after salinity stress (Fig 4d).

### Determination of transporter gene expression

**Fig. 5:**
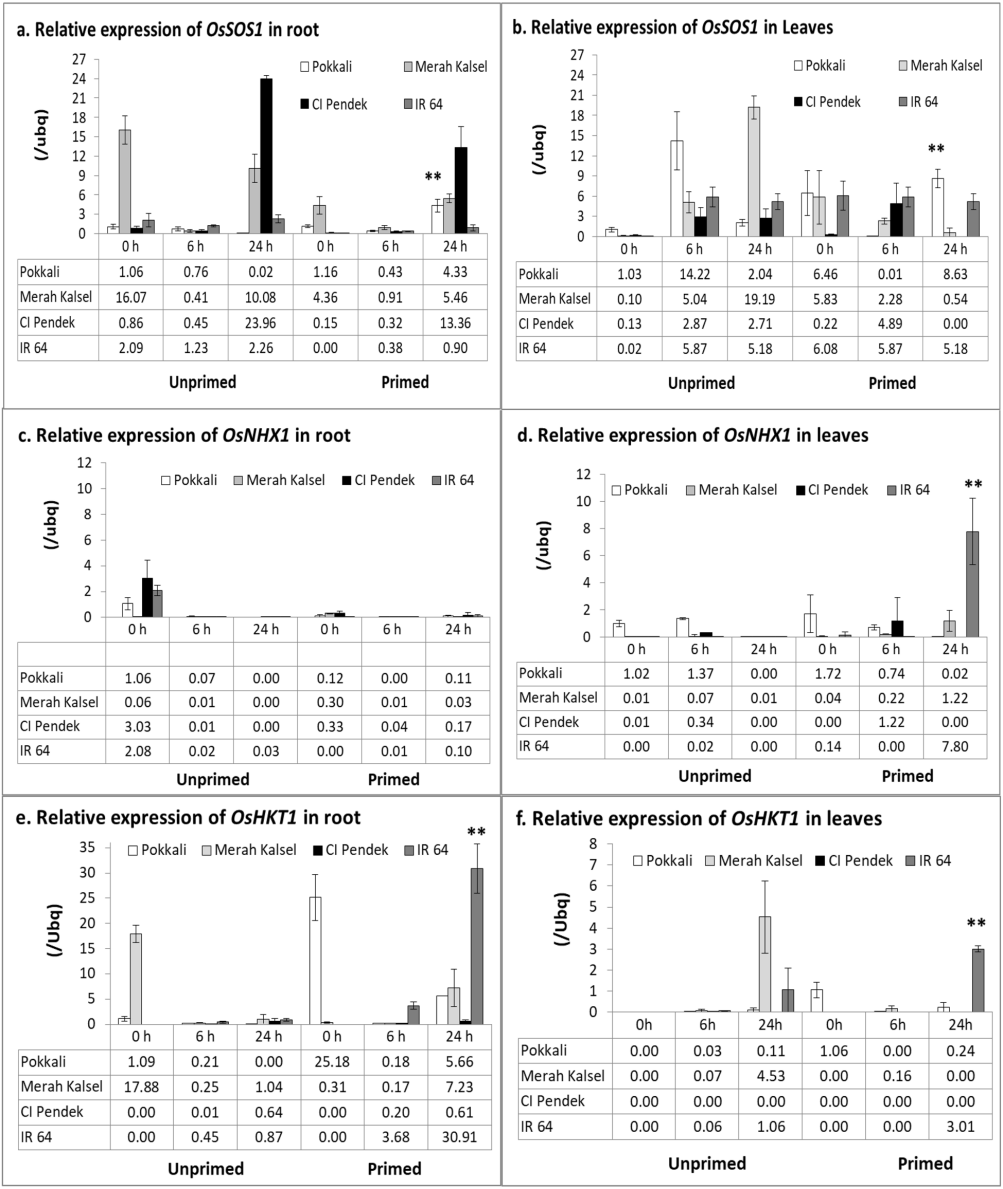
Relative expression of transporter genes in roots and leaves as compared with *UBIQUITIN*. Expression was measured at 0, 6, and 24 h after salinity treatment (200 mM NaCl). Values are means of three replicates ± SE

To understand the molecular basis of salinity stress with and without priming, gene expression was assessed using RT-qPCR. The expression of *OsNHX1* and *OsHKT1* in the roots and leaves of salinity sensitive rice, IR 64, was induced after seed priming treatment (Fig 5). In roots, the expression of *OsSOS1* increased in primed Pokkali, primed Merah Kalsel, and unprimed and primed CI Pendek after salinity stress (Fig 5a). In leaves, the expression of *OsSOS1* increased in primed Pokkali and unprimed Merah Kalsel Merah Kalsel after salinity stress (Fig 5b). The expression of *OsNHX1* increased in primed IR 64 leaves under saline conditions (Fig 5d). The expression of *OsHKT1* increased in primed Merah Kalsel and IR 64 roots under salinity stress (Fig 5e). The *OsHKT1* expression in unprimed Merah Kalsel and primed IR 64 leaves increased after salinity stress (Fig 5f).

## Discussion

Soil salinity is a global problem that reduces crop yields substantially. Maswada et al. (2018) estimated that ~ 10 million hectares of land are degraded annually. Seed priming improves plant growth, especially under unfavorable conditions (Farooq et al. 2005; Maswada et al. 2018). In this study, the effects of salinity stress on salinity-tolerant rice (Pokkali), salinity-susceptible rice (IR 64), and two pigmented rice cultivars, the salinity-tolerant CI Pendek and the moderately salinity-tolerant Merah Kalsel, with and without priming, were compared. The differences in the morpho-physiology and gene expression of these plants after salinity stress were also assessed in this study. The methods used in this study would be useful in attempts to improve the physiological characteristics of rice for agriculture. The salinity tolerance of Pokkali and Merah Kalsel, the moderately tolerant rice cultivars, increased slightly but the salinity tolerance in CI Pendek decreased after seed priming treatment. Seed priming of a salinity-susceptible rice cultivar, IR 64, greatly increased salinity tolerance.

The CC of leaves in each rice cultivar increased after seed priming treatment but then decreased after salt stress as compared with that of controls in both unprimed and primed seedlings. The enhancement of photosynthetic pigments under hydro- and halopriming in all three rice varieties points toward a role for seed priming in positively influencing the synthesis of chlorophylls and carotenoids during seedling growth. Seed priming in rice causes increases in chlorophyll and carotenoid contents under NaCl stress (Jamil et al. 2013). As a result of NaCl/polyethylene glycol stress, the photosynthetic pigment contents and the activity of photosystems decreased in all the varieties studied. These reductions may be due to the degradation of chlorophyll pigments or degradation of complexes involved in photosynthetic machinery (Jisha and Puthur 2014). According to Abd el-Samad et al. (2011), the reduction in CC under osmotic stress may be due to the suppression of enzymes required for chlorophyll synthesis or the destruction of chloroplasts and instability of the pigment protein complex.

Salinity causes cellular dehydration and induces increased solute concentrations in plants, thereby increasing the osmotic potential and leading to ion toxicity (Yang et al. 2018). Relative water content—the measure of water status in terms of cellular hydration as a consequence of leaf water potential and osmotic adjustment—normally decreases at higher salinity levels (Razzaque et al. 2019). The RWC decreases under salinity stress, possibly due to lower external (medium) water potential as compared with internal (tissue) water potential. The osmotic potential of leaves becomes more negative with increasing salinity stress (Maswada et al. 2018). In this study, the RWCs in primed seedlings were higher than those in unprimed seedlings under salinity conditions. Similarly, Djanaguiraman et al. (2006) observed that seed priming with *n*-Fe_2_O_3_ at 100 and 500 mg/L significantly increased the RWC (%) in rice leaves at 36 days after sowing (DAS), leading to turgor maintenance that results in salt tolerance improvement in rice. The mechanism involved in the maintenance of turgor, namely osmotic adjustment, is accumulation of compatible solutes (Maswada et al. 2018). The accumulation of compatible solutes is often considered a basic strategy for protection of plants from salinity, and the compatible solutes accumulate in the cytosol, contribute to the decrease of cytoplasmic water potential, and act as osmoprotectants (Reddy et al. 2017). Rice plants require a RWC content of more than 70% for healthy growth, while a RWC less than 60% is an indication of stress (Zhao et al. 2014). The salinity-susceptible rice (IR 64) was able to maintain a RWC value above 70% after seed halopriming treatment. This study confirmed that tolerant rice plants were able to maintain high RWC levels, while susceptible plants experienced a 15% reduction in RWC levels compared to control plants after 8 h of salt treatment (Ma et al. 2018). Growth (plant height, root length, and whole-plant dry weight) decreased under salinity stress (200 mM NaCl) compared with that of controls (0 mM NaCl), in both unprimed and primed seedlings. High salinity levels caused simultaneous reductions in seedling root and shoot dry biomass production (Razzaque et al. 2019). However, seed priming increased plant height, root length, and whole-plant dry weight in Merah Kalsel and IR 64 but not in salinity-tolerant rice. The increased growth and biomass associated with seed priming could be due to enhanced photosynthetic rates, photosystem II efficiency, water uptake, and decreased membrane damage (Maswada et al. 2018). The significant increases in chlorophyll content (*p* < 0.05; LSD), as observed in this study, might be due to the enhanced biomass.

To determine the mechanisms underlying salinity tolerance in rice, the expression profiles of the transporter genes encoding Na^+^ transport proteins were analyzed. The Na^+^/H^+^ antiporter, *OsSOS1*, localized in the plasma membrane, is considered a general regulator of Na^+^ export from cytosol (Shi et al. 2002). This study showed a higher level of induced expression of the *OsSOS1* gene in salinity-tolerant rice, which might be responsible for the relatively low Na^+^ accumulation in roots under salt stress. Salinity stress induced expression of the *OsSOS1* gene in leaves in salinity-tolerant rice and primed seedlings. Relative expression of the other Na^+^/H^+^ antiporter, *OsNHX1*, induced in primed seedling leaves under salinity stress, might be responsible for increased Na^+^ accumulation in the leaf vacuoles under salt stress. The Na^+^/H^+^ antiporter plays an important role in tolerance to salt stress by exchanging Na^+^ and H^+^ across the plasma or vacuolar membrane. The tonoplast Na^+^/H^+^ antiporter, which has been identified in several plant species, transports Na^+^ from the cytoplasm into vacuoles, thereby increasing the cytoplasmic K^+^/Na^+^ ratio and protecting cells from sodium toxicity (Fukuda et al. 1999). The functions of the *OsSOS1* and *OsNHX1* proteins are recognized as key determinants of salinity tolerance in higher plants. Furthermore, Na^+^ transporter, *OsHKT1*, is one of the main regulators of Na^+^ accumulation in shoots; this gene plays a role in the mechanism of exclusion of Na^+^ ions from shoots by recruiting Na^+^ ions from xylem and transporting them to xylem parenchyma cells in the root (Wangsawang et al. 2018). In this study, RT-qPCR analyses showed that priming seeds increased the relative expression of *OsNHX1* in the leaves and *OsHKT1* in the roots and leaves of salt-sensitive rice IR 64, which may improve salt tolerance via tissue tolerance mechanisms. Tissue tolerance, osmotic exclusion and ion exclusion prevent the accumulation of toxic concentrations of Na^+^ and Cl^−^ (Reddy et al. 2017).

Among salinity-tolerant traits in glycophytes, the most significant plant adaptation to salinity is the ability to restrict the transport and accumulation of Na^+^ in the leaf blades (Mekawy et al. 2015). Thus, seed priming increases Na^+^ concentrations in leaves, making them better able to handle salinity stress. This restricted transport of Na^+^ to the leaves is often accompanied by a reduced Na^+^/K^+^ ratio, which is relevant for the sustainability of normal metabolic functions (Tester and Davenport 2003). The other favorable trait, the maintenance of higher K^+^ concentrations in the leaves under both control and salinity stress conditions, was observed in salinity-tolerant rice. Maintenance of higher K^+^ concentrations, and thus lower Na^+^/K^+^ ratios in the tissues, is detrimental to the salinity tolerance of glycophytes because accumulation of Na^+^ in the cytosol disrupts K^+^-dependent biochemical reactions that are essential for plant growth. Earlier reports suggested that Ca^2+^ helps in the maintenance of cellular membrane integrity, thus reducing Na^+^ concentrations and favoring K^+^ absorption (Ashraf et al. 2003). Ca^2+^ also strongly influences the entry of Na^+^ into cells through high-affinity K^+^ carriers or through low-affinity channels called nonselective cation channels (Reddy et al. 2017). Decreased Na^+^ uptake and improved K^+^ uptake are among the important indicators of salinity tolerance (Wangsawang et al. 2018). The ability of plants to limit Na^+^ transport to shoots is important for the maintenance of growth rates and protection of the metabolic process in elongation cells from the toxic effects of Na^+^ (Razmjoo et al. 2008). Physiologically, the beneficial effects of these priming treatments can be attributed to increased accumulations of K^+^ with simultaneous decreases in Na^+^ uptake (Yang et al. 2018).

## Conclusion

Seed halopriming significantly increased the level of salinity tolerance in salinity-susceptible rice, IR 64, and moderately tolerant rice, Merah Kalsel. After seed priming treatment, IR 64 and Merah Kalsel seedlings survived under high salinity stress. Induction of expression of the *OsNHX1* and *OsHKT1* genes in susceptible rice, IR 64, after halopriming seed treatment balances the osmotic pressure and prevents the accumulation of toxic concentrations of Na^+^ via tissue tolerance mechanisms, resulting in plant tolerance to salinity stress. Seed halopriming decreased SES scores in the salinity-tolerant cultivars Pokkali and CI Pendek but did not affect their salinity tolerance.

## Data Availability

The datasets used and/or analysed during the current study are available from the corresponding author on reasonable request.

## Abbreviations

SES: Standard Evaluation System
*OsHKT1*: *Oryza sativa* High Affinity K + Transporter 1
*OsSOS1*: *Oryza sativa* Salt-Overly Sensitive 1
*OsNHX1*: *Oryza sativa* Na^+^/H^+^ antiporter 1
ECw: Electrical conductivity
RWC: Relative water content
RT-qPCR: Reverse transcription-quantitative polymerase chain reaction
NaCl: Sodium chloride
CaCl_2_: Calcium chloride
KCl: Potassium chloride
KNO_3_: Potassium nitrate
H_2_O_2_: Hydrogen peroxide
NH_4_NO_3_: Ammonium nitrate
NaH_2_PO_4_·2H_2_O: Sodium phosphate monobasic dihydrate
K_2_SO_4_: Potassium sulfate
MgSO_4_·7H_2_O: Magnesium sulfate heptahydrate
MnCl·4 H_2_O: Manganese (II) chloride tetrahydrate
(NH_4_)_6_·MO_7_O_24_·4H_2_O: Molybdic acid ammonium salt tetrahydrate
H_3_BO_3_: Boric acid
ZnSO_4_·7H_2_O: Zinc sulfate heptahydrate
CuSO_4_·5H_2_O: Copper sulfate pentahydrate
FeCl_3_·6H_2_O: Iron(III) chloride hexahydrate
C_6_H_8_O_7_·H_2_O: Citric acid monohydrate
H_2_SO_4_: Sulfuric acid
NaOH: Sodium hydroxide
